# Sequencing results from multiple individuals of different ethnicities strongly question the existence of the *KCNE1B* pseudogene

**DOI:** 10.1038/s41431-019-0502-6

**Published:** 2019-09-16

**Authors:** Marta Diñeiro, Guadalupe A. Cifuentes, Raquel Capín, Adrián Santiago, Andrea Otero, David Castillo, Patricia C. Pruneda, Gonzalo R. Ordóñez, Rubén Cabanillas, Juan Cadiñanos

**Affiliations:** 1 Instituto de Medicina Oncológica y Molecular de Asturias (IMOMA) S. A., Oviedo, Spain; 2 DREAMgenics S. L., Oviedo, Spain

**Keywords:** Genome, Genetic testing

## To the Editor:

In regard to the Viewpoint by Pantou et al. “The potential presence of the highly similar paralogue gene *KCNE1B* blurs the genetic basis of *KCNE1*-LQTS patients” [1], we have collected the following evidence in our laboratory, strongly supporting the possibility that the pseudogene *KCNE1B*, absent from the GRCh37 version of the human genome, is an artifact introduced in GRCh38:

- We have evaluated the genotype of the rs1805127 SNP on 58 deaf patients analyzed with the OTOgenics Next Generation Sequencing (NGS) capture panel [2]. This SNP was selected because the reference nucleotide for *KCNE1* (T) is different from the reference nucleotide for the paralogue position of the *KCNE1B* pseudogene (C), according to GRCh38 (Supplementary Fig. 1). Considering together all reads potentially originating from *KCNE1* or *KCNE1B*, all patients show variant reads/total reads ratios of 0, 0.5 or 1, as it would be expected for the existence of a single locus with two alleles, instead of two paralogue loci with four alleles. If *KCNE1B* was not an artifact, since the sequences surrounding rs1805127 are identical between *KCNE1* and the corresponding paralogue *KCNE1B* region, reads originating from all four alleles would be mapped together and *KCNE1* heterozygous patients with a ratio of 0.25 should be identified (Supplementary Table 1).
- We have evaluated a daughter-father-mother trio in which the father and the daughter are heterozygous for the rs74315445 *KCNE1* SNV, while the mother is wild type. PCR was performed using primers that, according to GRCh38, should not be able to distinguish between *KCNE1* and *KCNE1B* (Supplementary Fig. 1). Sanger sequencing of the obtained PCR products shows heterozygous peaks with identical heights for both alleles from the father’s and the daughter’s DNAs, which is not compatible with a variant affecting only one of four alleles. Although the father could be homozygous for the variant in the *KCNE1* locus (which could explain the similar height of both peaks in his sample if *KCNE1B* was real), the mother is wild type and, therefore, the daughter could not possibly inherit two variant alleles from her parents (Supplementary Fig. 2).
- We have PCR amplified the regions containing the positions for rs1805127 and rs1805128 SNPs on DNA from lymphoblastoid cell lines from ten individuals representing ten different ethnic origins from four continents in the same conditions unable to distinguish *KCNE1* from *KCNE1B*. In all cases, Sanger sequencing of the PCR product shows clear wild type or heterozygous genotypes for rs1805127 (with peaks of identical heights for the alternative and reference *KCNE1* alleles in heterozygotes) and homozygous *KCNE1* reference (C/C) genotypes for rs1805128, with no sign of the reference *KCNE1B* (T) allele (Supplementary Table 2 and Supplementary Fig. 3).

In our opinion, these data, together with those presented by Pantou et al., strongly suggest that *KCNE1B* is an artifact that should be reviewed and curated. This would reduce the probabilities of false negative results caused by misalignment of variant reads originating from the true and clinically relevant *KCNE1* locus to the *KCNE1B* region of the GRCh38 reference genome.

## Supplementary information

Supplementary Materials and Methods

Supplementary Table 1

Supplementary Table 2

Supplementary Figure 1

Supplementary Figure 2

Supplementary Figure 3
