# Supplementary material for: Sequencing results from multiple individuals of different ethnicities strongly question the existence of the KCNE1B pseudogene
Source: Eur J Hum Genet. 2019 Sep 16;28(4):401–2. doi: 10.1038/s41431-019-0502-6 (PMC7080829; doi:10.1038/s41431-019-0502-6)
Supplement: Supplementary file 1 — Supplementary Materials and Methods [file 41431_2019_502_MOESM1_ESM.docx]

**Supplementary Material and Methods**

**DNA extraction and NGS**

DNA extractions from patient blood and Coriell lymphoblastoid cell lines, as well as NGS, were performed as described in Cabanillas *et al.*^2^

**PCR and Sanger Sequencing**

Primers designed to amplify both a 409 bp genomic region containing the rs74315445, rs1805128 and rs1805127 *KCNE1* SNPs and the putative paralogue *KCNE1B* region are shown below:

| **Oligo ID** | **Sequence** |
| --- | --- |
| OL-1842 | TTCATGGGGAAGGCTTCGTC |
| OL-2654 | GGAACCTTAATGCCCAGGAT |

Amplifications were performed using 12.5 pmol of each primer, 50 ng of germline DNA as template and 5 μl of Megamix Double premix (Microzone, UK), with the following PCR program: 95 °C for 5 min; 30 cycles of 95 °C for 1 min, 58 °C for 1 min and 72 °C for 1 min; and a final step of 72 °C for 10 min. PCR products were purified using ExoBAP (EURx, Poland) and sequenced using primers OL-1842 and OL-2654 in separate reactions, with the Big Dye Terminator v.3.1 Cycle Sequencing kit (Applied Biosystems, USA) on an Applied Biosystems 3130XL Genetic Analyzer.
