# Supplementary material for: Sequencing results from multiple individuals of different ethnicities strongly question the existence of the KCNE1B pseudogene
Source: Eur J Hum Genet. 2019 Sep 16;28(4):401–2. doi: 10.1038/s41431-019-0502-6 (PMC7080829; doi:10.1038/s41431-019-0502-6)
Supplement: Supplementary file 2 — Supplementary Table 1 [file 41431_2019_502_MOESM2_ESM.pdf]

Supplementary Table 1. Total reads, alternative allele reads and alternative allele frequencies of the rs1805127 SNP considering all reads potentially generated from *KCNE1* and *KCNE1B* of 58 gDNA samples analyzed with the OTOgenics NGS panel

gDNA Sample ID	Total reads	Alternative reads	Alternative allele frequency
B02611	717	715	0.997
B02617	694	334	0.481
B02621	898	424	0.472
B02637	412	217	0.527
B02669	1014	1010	0.996
B02741	533	530	0.994
B02743	478	241	0.504
B02744	592	280	0.473
B02745	489	227	0.464
B02747	814	413	0.507
B02786	308	308	1
B02805	715	0	0
B02812	1058	529	0.5
B02815	972	972	1
B02816	637	317	0.498
B02819	1299	1299	1
B02832	1046	1045	0.999
B02879	780	778	0.997
B02880	526	260	0.494
P00288	1208	q	0.001
P00292	605	264	0.436
P00379	1003	996	0.993
P00500	687	339	0.493
P00509	640	636	0.994
P00536	532	0	0
P00548	420	207	0.493
P00566	966	483	0.5
P00733	633	302	0.477
P00737	1046	1	0.001
P00746	289	137	0.474
P00815	807	801	0.993
P00843	712	710	0.997
P00913	931	430	0.462
P00946	1389	678	0.488
P00969	852	851	0.999
P01022	762	749	0.983
P01052	904	0	0
P01949	1341	1341	1
P02694	500	253	0.506
P02695	777	777	1
P02696	1143	563	0.493
P02697	807	0	0
P02699	1094	519	0.474
P02720	1273	634	0.498
P02706	910	451	0.496
P02743	895	894	0.999
P02744	875	874	0.999
P02745	949	495	0.522
P02746	1080	510	0.472
P02747	756	358	0.474
P02748	801	801	1
P02749	1367	1367	1
P02750	1207	1207	1
P02759	837	0	0
P02767	629	314	0.499
P02776	1183	1182	0.999
P02777	980	979	0.999
P02787	904	904	1
