# Supplementary material for: Sequencing results from multiple individuals of different ethnicities strongly question the existence of the KCNE1B pseudogene
Source: Eur J Hum Genet. 2019 Sep 16;28(4):401–2. doi: 10.1038/s41431-019-0502-6 (PMC7080829; doi:10.1038/s41431-019-0502-6)
Supplement: Supplementary file 3 — Supplementary Table 2 [file 41431_2019_502_MOESM3_ESM.pdf]

Supplementary Table 2. Summary of Sanger sequencing results of a PCR product designed to amplify both the genomic region surrounding the rs1805128 and rs1805127 SNPs of *KCNE1* and its putative paralogue *KCNE1B* region (see also Supp. Fig. 3)

gDNA Sample ID	Coriell Cell line ID	HapMap/1000 Genomes Sample ID	Population of origin	Summary of observed Sanger sequencing peaks	
				rs1805128 (C: REF in *KCNE1*; T: REF in *KCNE1*)	rs1805127 (T: REF in *KCNE1*; C: ALT in *KCNE1* and REF in *KCNE1B*)
I00063	GM19020	NA19020	Luhya in Webuye, Kenya, African Ancestry	C (no residual T)	T (no residual C)
I00064	GM18486	NA18486	Yoruba in Ibadan, Nigeria, African Ancestry	C (no residual T)	T/C (equal peak heights)
I00065	GM20298	NA20298	African Ancestry in Southwest US	C (no residual T)	T/C (equal peak heights)
I00056	GM19794	NA19794	Mexican Ancestry in Los Angeles, California, American Ancestry	C (no residual T)	T/C (equal peak heights)
I00066	HG00740	HG00740	Puerto Rican in Puerto Rico, American Ancestry	C (no residual T)	T/C (equal peak heights)
I00067	GM18570	NA18570	Han Chinese in Beijing, China, East Asian Ancestry	C (no residual T)	T (no residual C)
I00062	GM18960	NA18960	Japanese in Tokyo, Japan, East Asian Ancestry	C (no residual T)	T/C (equal peak heights)
I00060	HG00110	HG00110	British in England and Scotland	C (no residual T)	T (no residual C)
I00068	HG00320	HG00320	Finnish in Finland, European Ancestry	C (no residual T)	T/C (equal peak heights)
I00069	GM12872	NA12872	Utah residents (CEPH) with Northern and Western European ancestry	C (no residual T)	T/C (equal peak heights)
