# Supplementary material for: Sequencing results from multiple individuals of different ethnicities strongly question the existence of the KCNE1B pseudogene
Source: Eur J Hum Genet. 2019 Sep 16;28(4):401–2. doi: 10.1038/s41431-019-0502-6 (PMC7080829; doi:10.1038/s41431-019-0502-6)
Supplement: Supplementary file 4 — Supplementary Figure 1 [file 41431_2019_502_MOESM4_ESM.pdf]

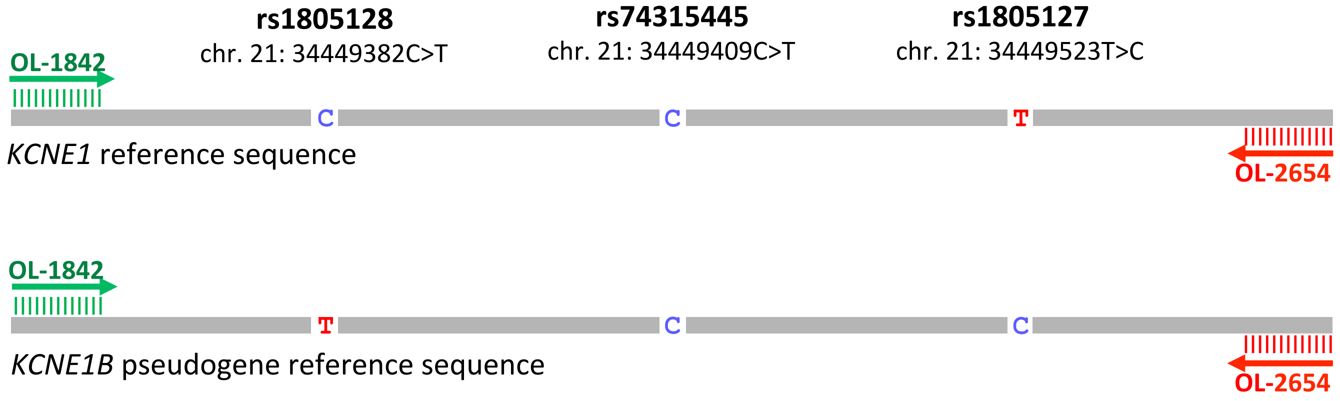

Supplementary Figure 1. Schematic representation of the GRCh38 reference sequences of the genomic regions of the *KCNE1* gene (top) and the *KCNE1B* pseudogene (bottom) evaluated in this work. Gray bars represent identical paralogous sequences. The positions of the primers used to amplify the region studied by Sanger sequencing (OL-1842 and OL-2654) and the SNPs contained within it (rs1805128, rs73415455 and rs1805127) are shown. Genomic coordinates indicated for the SNPs correspond to those of *KCNE1* according to GRCh38.
