# Supplementary material for: Sequencing results from multiple individuals of different ethnicities strongly question the existence of the KCNE1B pseudogene
Source: Eur J Hum Genet. 2019 Sep 16;28(4):401–2. doi: 10.1038/s41431-019-0502-6 (PMC7080829; doi:10.1038/s41431-019-0502-6)
Supplement: Supplementary file 5 — Supplementary Figure 2 [file 41431_2019_502_MOESM5_ESM.pdf]

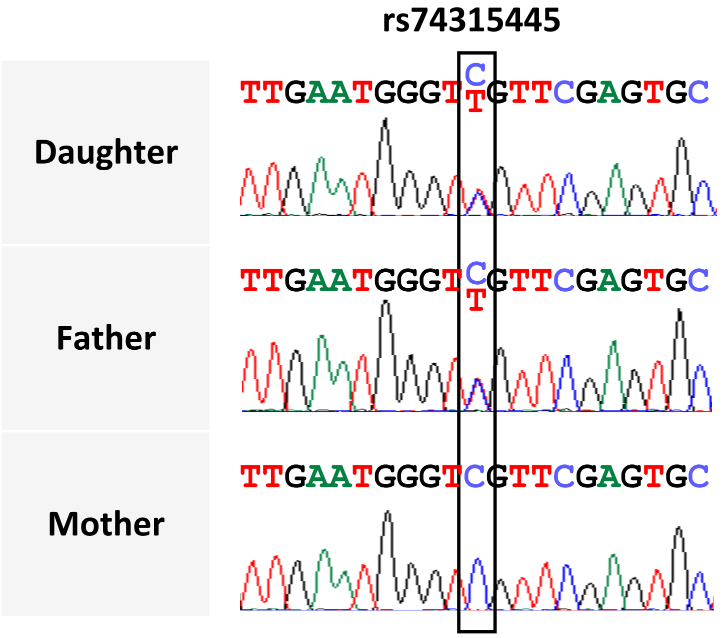

Supplementary Figure 2. Genotypes and Sanger sequencing electropherograms showing allele peak heights for the rs74315445 SNP in gDNA samples from a heterozygous individual and her parents
