# Supplementary material for: Sequencing results from multiple individuals of different ethnicities strongly question the existence of the KCNE1B pseudogene
Source: Eur J Hum Genet. 2019 Sep 16;28(4):401–2. doi: 10.1038/s41431-019-0502-6 (PMC7080829; doi:10.1038/s41431-019-0502-6)
Supplement: Supplementary file 6 — Supplementary Figure 3 [file 41431_2019_502_MOESM6_ESM.pdf]

NA19020	Luhya in Webuye, Kenya,<br>**African Ancestry**	
NA18486	Yoruba in Ibadan, Nigeria,<br>**African Ancestry**	
NA20298	**African Ancestry**<br>in Southwest US	
NA19794	Mexican Ancestry in Los Angeles, California,<br>**American Ancestry**	
HG00740	Puerto Rican in Puerto Rico,<br>**American Ancestry**	
NA18570	Han Chinese in Beijing, China,<br>**East Asian Ancestry**	
NA18960	Japanese in Tokyo, Japan,<br>**East Asian Ancestry**	
HG00110	British in England and Scotland,<br>**European Ancestry**	
HG00320	Finnish in Finland,<br>**European Ancestry**	
NA12872	Utah residents (CEPH) with Northern and Western,<br>**European Ancestry**	

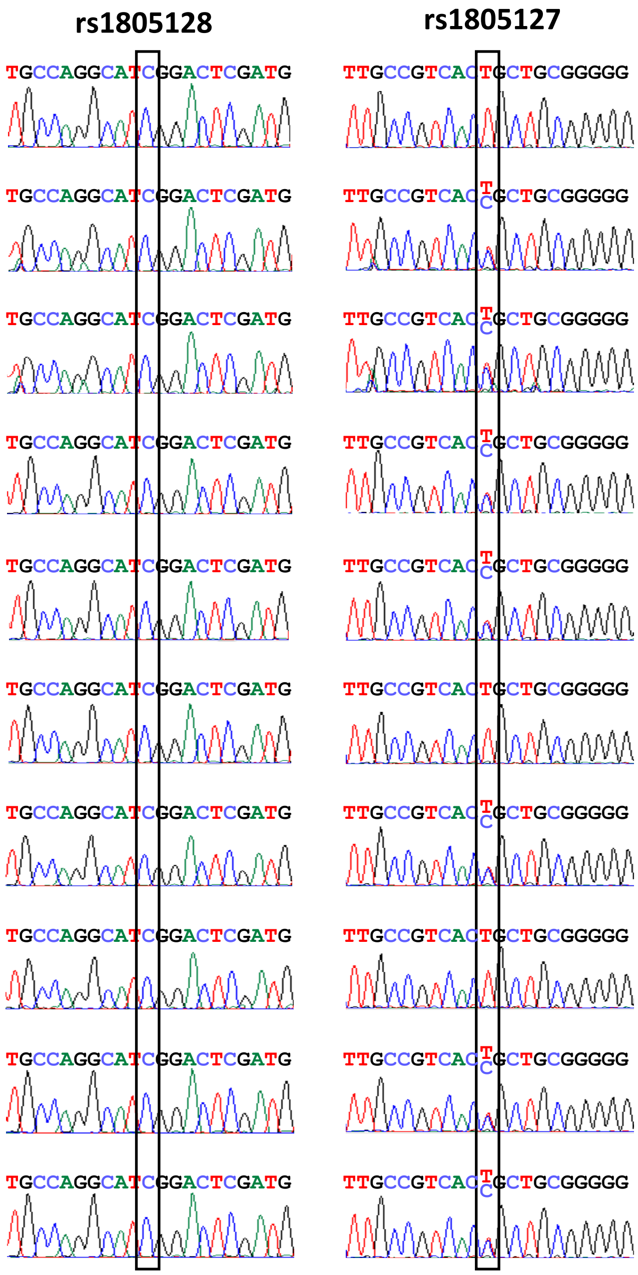

Supplementary Figure 3. Genotypes and Sanger sequencing electropherograms showing allele peak heights for the rs1805128 and rs1805127 SNPs in gDNA samples from individuals of10 different global ethnicities. 1000 Genomes / HapMap project sample IDs are shown on the left column

Please, insert a space between "of" and "10 different global ethnicities"
